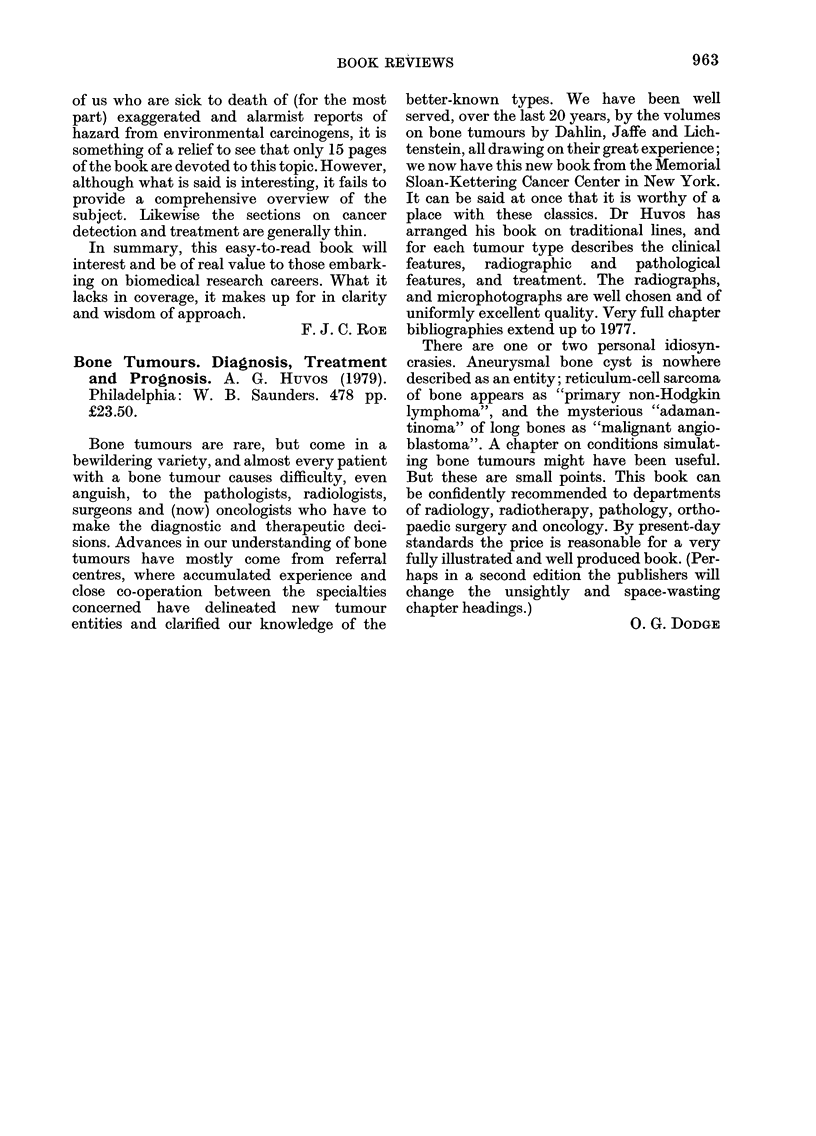# Bone Tumours. Diagnosis, Treatment and Prognosis

**Published:** 1979-12

**Authors:** O. G. Dodge


**Bone Tumours. Diagnosis, Treatment and Prognosis.** A. G. Huvos (1979). Philadelphia: W. B. Saunders. 478 pp. £23.50.

Bone tumours are rare, but come in a bewildering variety, and almost every patient with a bone tumour causes difficulty, even anguish, to the pathologists, radiologists, surgeons and (now) oncologists who have to make the diagnostic and therapeutic decisions. Advances in our understanding of bone tumours have mostly come from referral centres, where accumulated experience and close co-operation between the specialties concerned have delineated new tumour entities and clarified our knowledge of the better-known types. We have been well served, over the last 20 years, by the volumes on bone tumours by Dahlin, Jaffe and Lichtenstein, all drawing on their great experience; we now have this new book from the Memorial Sloan-Kettering Cancer Center in New York. It can be said at once that it is worthy of a place with these classics. Dr Huvos has arranged his book on traditional lines, and for each tumour type describes the clinical features, radiographic and pathological features, and treatment. The radiographs, and microphotographs are well chosen and of uniformly excellent quality. Very full chapter bibliographies extend up to 1977.

There are one or two personal idiosyncrasies. Aneurysmal bone cyst is nowhere described as an entity; reticulum-cell sarcoma of bone appears as "primary non-Hodgkin lymphoma", and the mysterious "adamantinoma" of long bones as "malignant angioblastoma". A chapter on conditions simulating bone tumours might have been useful. But these are small points. This book can be confidently recommended to departments of radiology, radiotherapy, pathology, orthopaedic surgery and oncology. By present-day standards the price is reasonable for a very fully illustrated and well produced book. (Perhaps in a second edition the publishers will change the unsightly and space-wasting chapter headings.)

O. G. Dodge